# Supplementary material for: Capacitation promotes a shift in energy metabolism in murine sperm
Source: Front Cell Dev Biol. 2022 Aug 23;10:950979. doi: 10.3389/fcell.2022.950979 (PMC9445201; doi:10.3389/fcell.2022.950979)
Supplement: Supplementary file 1 [file DataSheet2.PDF]

Figure S2

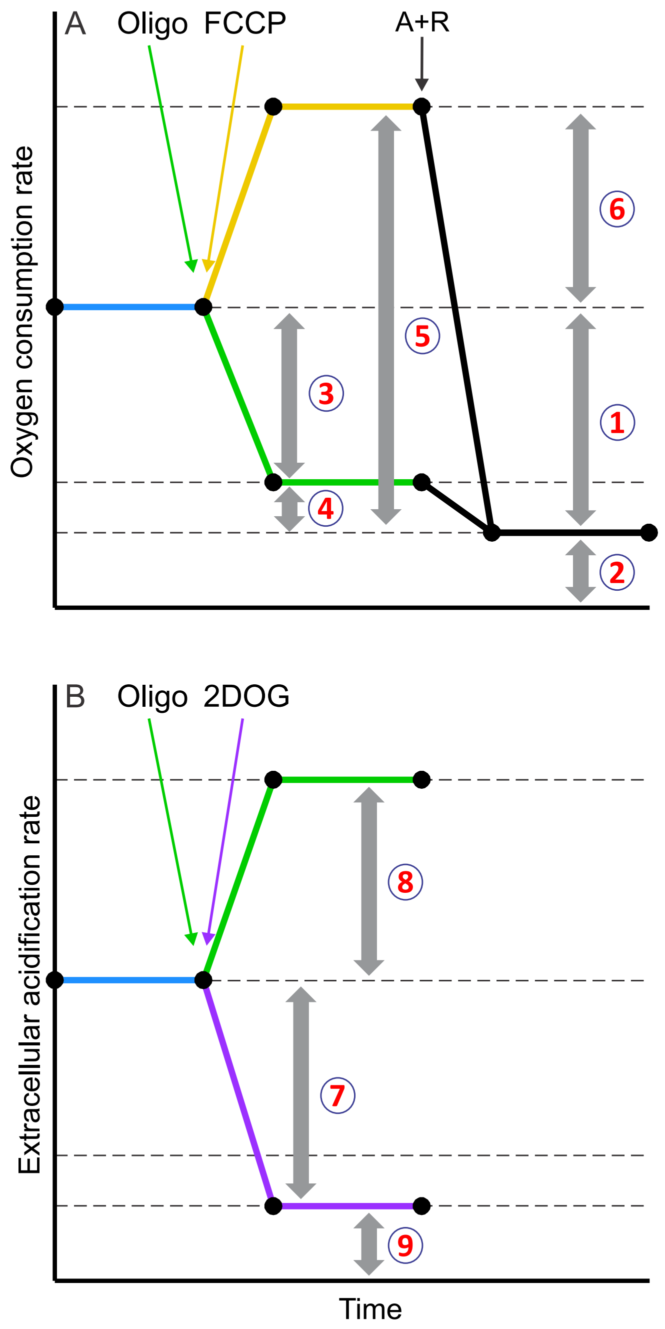

Supplementary Figure S2. Metabolic parameters calculated to assess sperm bioenergetic phenotype. The rates of oxygen consumption **(A)** and extracellular acidification **(B)** are monitored while alternative metabolic effectors are added. Oligo: 5 µM oligomycin is added to inhibit mitochondrial ATP synthesis; FCCP: 1 µM FCCP (respiration uncoupler) is added to ensure the maximum rate of respiration is reached; A+R: simultaneous addition of 1 µM antimycin and 1 µM rotenone to inhibit mitochondrial respiration; 2DOG: addition of 50 mM 2-deoxy-glucose to inhibit glycolytic ATP and pyruvate production. The following bioenergetic parameters are calculated for the rates of oxygen consumption and extracellular acidification indicated in the scheme: 1) Basal respiration rate; 2) Non-mitochondrial oxygen consumption; 3) Respiratory ATP production; 4) Proton leak; 5) Maximum respiration rate; 6) Spare respiratory capacity; 7) Basal glycolysis rate; 8) Glycolytic reserve; 9) Non-glycolytic acidification.
