# Supplementary material for: Capacitation promotes a shift in energy metabolism in murine sperm
Source: Front Cell Dev Biol. 2022 Aug 23;10:950979. doi: 10.3389/fcell.2022.950979 (PMC9445201; doi:10.3389/fcell.2022.950979)
Supplement: Supplementary file 2 [file Table1.DOCX]

**Supplementary Table S1.** Calculation of sperm metabolic parameters based on OCR and ECAR values obtained from extracellular flux analyses. Each parameter is the result of subtracting the values under condition B from the values under condition A. ^a^Calculated only in experiments treated with oligomycin, antimycin and rotenone. ^b^Calculated only in experiments treated with FCCP. ^c^Calculated only in experiments treated with 2DOG.

| **Parameter** | **Condition A** | **Condition B** |
| --- | --- | --- |
| Basal respiration rate^a^ | OCR before any additions | OCR after the addition of antimycin A and rotenone |
|  |  |  |
| Respiratory ATP production^a^ | OCR before any additions | OCR after the addition of oligomycin |
|  |  |  |
| Proton leak^a^ | OCR after the addition of oligomycin | OCR after the addition of antimycin A and rotenone |
|  |  |  |
| Maximum respiration rate^b^ | OCR after the addition of FCCP | OCR after the addition of antimycin A and rotenone |
|  |  |  |
| Spare respiratory capacity^b^ | OCR after the addition of FCCP | OCR before any additions |
|  |  |  |
| Basal glycolysis rate^c^ | ECAR before any additions | ECAR after the addition of 2DOG |
|  |  |  |
| Glycolytic reserve^a^ | ECAR after the addition of oligomycin | ECAR before any additions |
