# Supplementary material for: Capacitation promotes a shift in energy metabolism in murine sperm
Source: Front Cell Dev Biol. 2022 Aug 23;10:950979. doi: 10.3389/fcell.2022.950979 (PMC9445201; doi:10.3389/fcell.2022.950979)
Supplement: Supplementary file 3 [file DataSheet3.PDF]

Figure S3

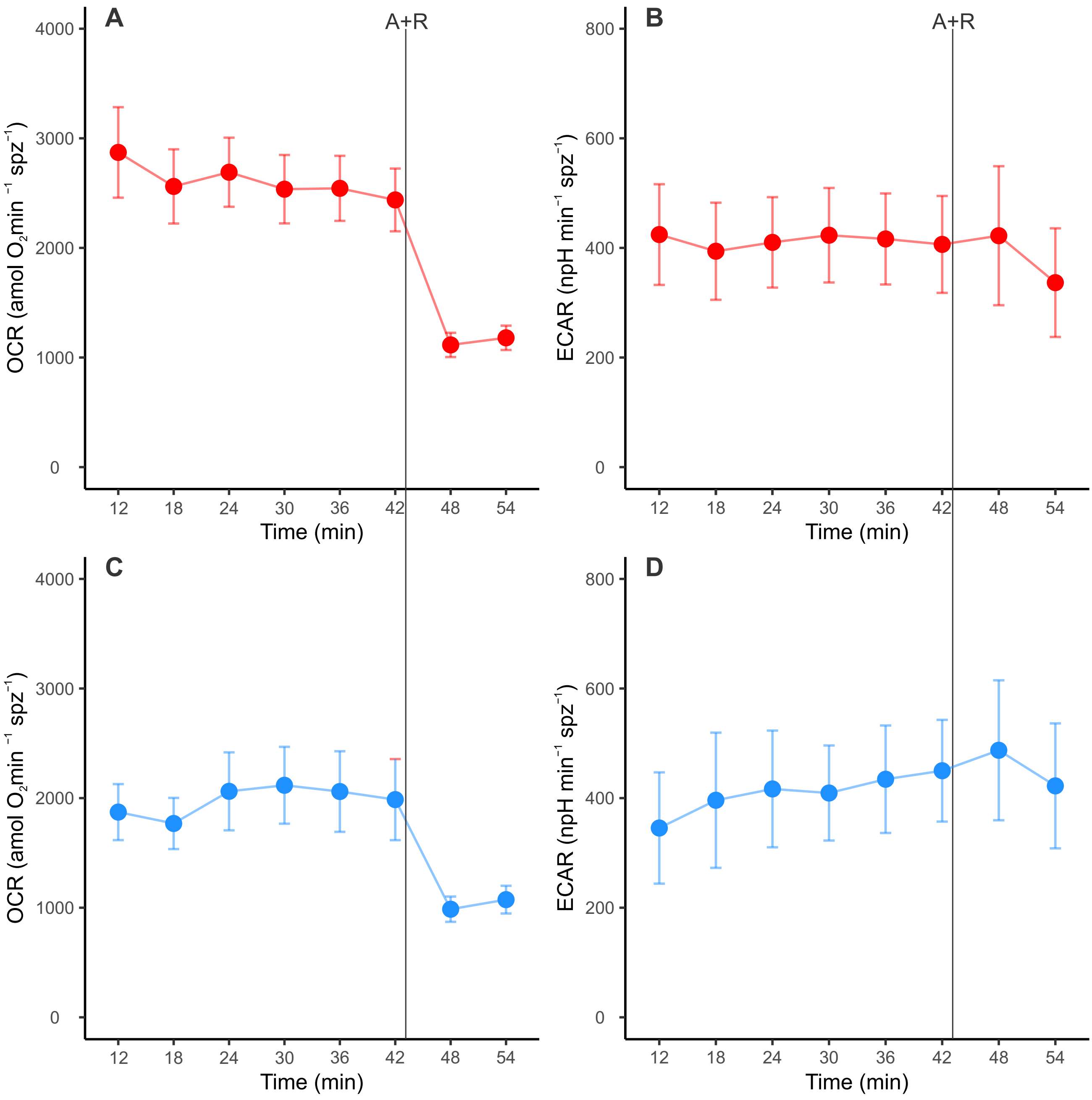

Supplementary Figure S3. Real-time measurements of oxygen consumption rate (OCR **(A, C)** and extracellular acidification rate (ECAR) **(B, D)** in mouse sperm. Sperm were incubated for 1 hour in non-capacitating **(A, B** - red symbols**)** and capacitating **(C, D** - blue symbols**)** conditions prior to extracellular flux analysis. Values have been normalized by sperm numbers inside each well. Symbols and whiskers correspond to mean ± standard error. Time = 0 was defined as the start of the 1st measurement cycle; measurement cycles 3 to 10 are reported. The line labeled as “A+R” marks the addition of 1 µM antimycin + 1 µM rotenone.
