# Supplementary material for: Capacitation promotes a shift in energy metabolism in murine sperm
Source: Front Cell Dev Biol. 2022 Aug 23;10:950979. doi: 10.3389/fcell.2022.950979 (PMC9445201; doi:10.3389/fcell.2022.950979)
Supplement: Supplementary file 4 [file DataSheet1.PDF]

Figure S1

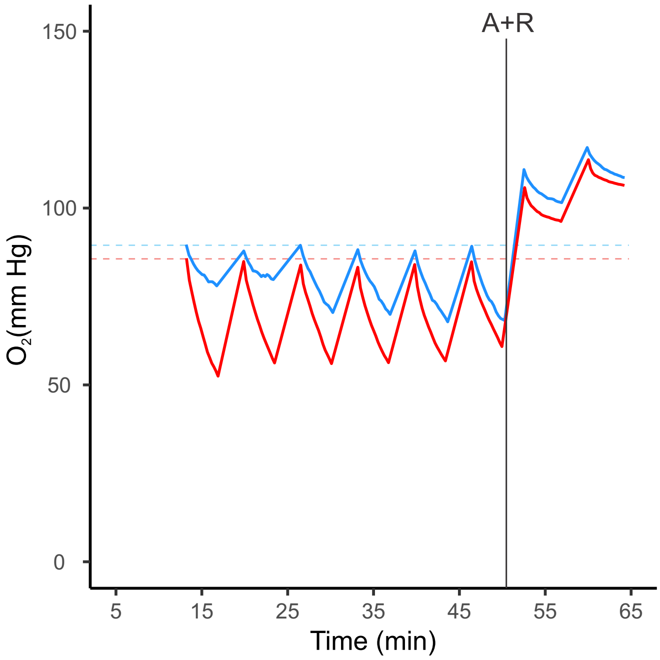

Supplementary Figure S1. Real-time measurement of oxygen levels during extracellular flux analysis using free-swimming mouse sperm. Each oxygen curve corresponds to one well of a Seahorse Xfp microplate during a representative experiment. When the sensor cartridge is lowered, forming a closed microchamber between the bottom of the plate and the cartridge, oxygen is consumed by the contained cell population (decreasing phase) and the rate of consumption is estimated by the analysis software. After measurement, the cartridge is elevated and the content of the wells is mixed, leading to the recovery of previous oxygen levels (increasing phase). Sperm were incubated in non-capacitating (red line) or capacitated (blue line) conditions for 1h previous to the experiment. Dashed horizontal lines: oxygen level at the beginning of the first measurement. Black vertical line: addition of 1 µM antimycin and 1 µM rotenone.
